# Approaching Optimal pH Enzyme Prediction with Large Language Models

**DOI:** 10.1021/acssynbio.4c00465

**Published:** 2024-08-28

**Authors:** Mark Zaretckii, Pavel Buslaev, Igor Kozlovskii, Alexander Morozov, Petr Popov

**Affiliations:** † Tetra D AG, Shaffhausen 8200, Switzerland; ‡ Constructor University Bremen gGmbH, Bremen 28759, Germany; § Nanoscience Center and Department of Chemistry, University of Jyväskylä, Jyväskylä 40014, Finland; ∥ Independent researcher, Moscow 119991, Russia; ⊥ Constructor Technology AG, Shaffhausen 8200, Switzerland

**Keywords:** protein engineering, enzyme optimal pH, large language models, machine learning

## Abstract

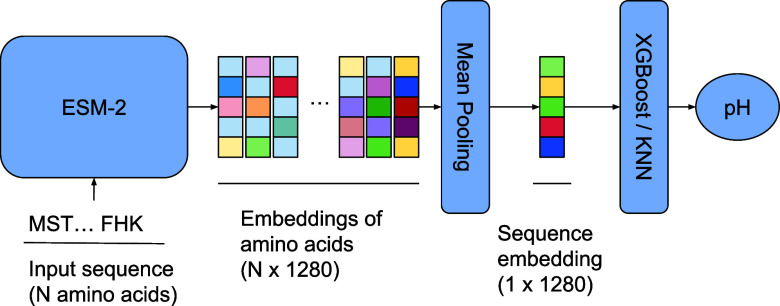

Enzymes are widely used in biotechnology due to their ability to catalyze chemical reactions: food making, laundry, pharmaceutics, textile, brewing—all these areas benefit from utilizing various enzymes. Proton concentration (pH) is one of the key factors that define the enzyme functioning and efficiency. Usually there is only a narrow range of pH values where the enzyme is active. This is a common problem in biotechnology to design an enzyme with optimal activity in a given pH range. A large part of this task can be completed *in silico*, by predicting the optimal pH of designed candidates. The success of such computational methods critically depends on the available data. In this study, we developed a language-model-based approach to predict the optimal pH range from the enzyme sequence. We used different splitting strategies based on sequence similarity, protein family annotation, and enzyme classification to validate the robustness of the proposed approach. The derived machine-learning models demonstrated high accuracy across proteins from different protein families and proteins with lower sequence similarities compared with the training set. The proposed method is fast enough for the high-throughput virtual exploration of protein space for the search for sequences with desired optimal pH levels.

## Introduction

Enzymes are catalytic molecules that are widely used in biotechnological production: food, brewing, fermentation, textile, laundry, paper, and pharmaceutical industries rely on enzymes.^1^ Commonly, the catalytic reactions start by transferring the proton from a protein residue to the substrate, forming the stable charged intermediate.^2−5^ The need for such proton transfer requires the amino acids forming the active site to be in a particular protonation state, which is defined by the solution proton concentration (pH) and the amino acid proton affinity (p*K*_a_). Despite p*K*_a_ values being well-known for single amino acids in water,^6^ the electrostatic interactions formed by the protein environment can lead to significant p*K*_a_ shifts. These shifts are usually unknown, making it difficult to predict the pH range for which the reaction can be catalyzed by enzymes.

To close the gap in experimental knowledge of amino acid p*K*_a_ values in proteins, a lot of computational tools have been developed aiming to predict those values.^7−12^ However, there is still a need for improved methods for the p*K*_a_ prediction.^13^ Most of the first-principles methods require a structural model to predict p*K*_a_. However, biotechnology often requires to design of a novel enzyme working in the given pH range without prior information about the atomic structure. One can use algorithms for protein structure prediction,^14−16^ to create a structural model for p*K*_a_ prediction of protein amino acids. However, structural models might be computationally costly, preventing such workflows from being applied for screening large databases of enzyme candidates, let alone the methods for p*K*_a_ predictions still work better for the experimentally determined structures.^13^ Additionally, due to possible interactions between the amino acids, derivation of optimal pH range from individual p*K*_a_ is not always evident.^17,18^ Thus, to facilitate the design of new enzymes for biotechnological production, new fast methods for predicting optimal enzyme pH range from its sequence are in high demand.

In contrast to structural methods for p*K*_a_ prediction, the majority of sequence-based approaches are knowledge-based. The models are first trained on experimental data sets that contain information about both enzyme sequence and optimal pH and then applied to new sequences to predict their properties. One of the first machine learning models solved classification problems, for example, to discriminate between the alkaline (active within pH > 7.0) and acidic (active within pH < 7.0) enzymes.^19−22^ The other models relied on neural networks to predict optimal pH for enzymes from a specific protein family, such as beta-glucosidase^23^ or glucoside-hydrolase.^24^ Finally, nonspecific machine learning methods emerge that rely on the protein embeddings calculated with large language models.^25^ The applicability of knowledge-based methods depends on the size and quality of the training data set. Most of the existing models utilized either manually prepared databases, or publicly available databases such as Brenda-Enzymes.^26,27^ In this study, we present a novel machine learning method that predicts the optimal pH range of enzymes solely based on their amino acid sequence. We rigorously evaluated the performance of our developed method using various train-validation splitting strategies, consistently observing robust and reliable predictions. Furthermore, our approach exhibits the potential for continuous improvement through the incorporation of new data into the training process. This indicates that as new enzyme pH data become available, our method can be enhanced to achieve even higher accuracy and predictive power. The developed method, dubbed OphPred, is fast in both the learning and inference stages, allowing efficient screenings of a thousand enzymes in less than a second, making it highly practical for large-scale analysis and screening tasks. Finally, to ensure widespread accessibility and usability, we have implemented our method in a user-friendly, zero-code platform that can be easily accessed and utilized by the scientific community.

## Results and Discussion

**Figure 1:**
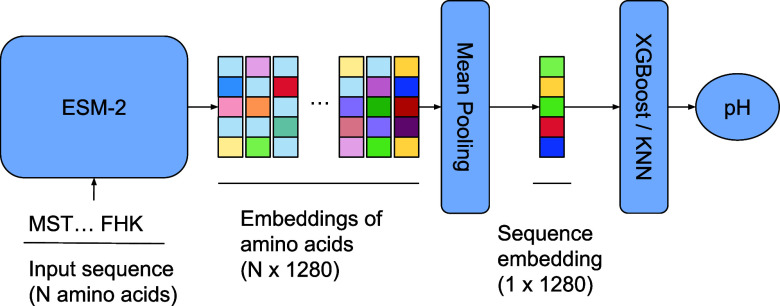
Illustration of the model pipeline.

Here, we present OphPred, the sequence- and machine learning-based approach to predict the optimal pH of a protein (see Figure 1). OphPred utilizes the ESM-2 protein language model in combination with KNN and XGBoost models. It is trained on the Brenda-Enzymes data set. We used rigorous validation involving four different splitting strategies: random, homology based, PFAM based, and EC based to avoid bias related to the sharing of similar sequences between the training and validation sets. Given the train-validation split, we processed protein sequences using the ESM-2 protein language model followed by the derivation of k-nearest neighbor (KNN) and eXtreme gradient boosting (XGBoost) models to predict enzyme optimal pH. To train the models, we used the Brenda-Enzymes data sets of optimal pH values collected for ∼3,000 (version November 2021) and for ∼10,000 (version March 2023) proteins with the UniProt identifiers. Hereinafter, we provide the results obtained for the models trained with the enrichment from the newer version of the Brenda-Enzymes data set (see Methods), while the corresponding results for the models trained using the older version are provided in the Supporting Information.

### Random and Homology Split

**Figure 2:**
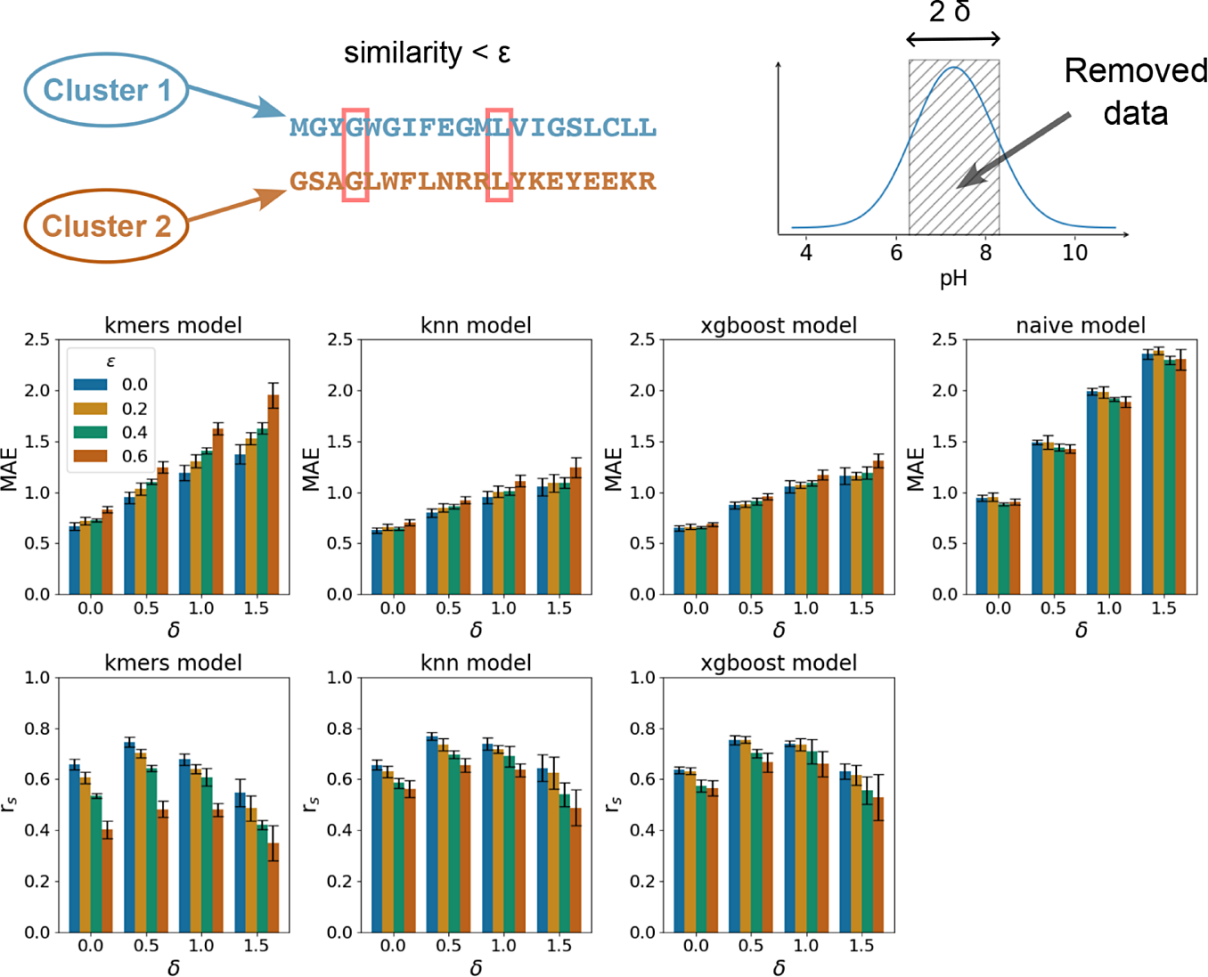
Performance of different models trained on random (ϵ = 0) and homology splits. The top row schematically explains the meaning of the ϵ and δ parameters. The middle row shows MAE metrics for different models and combinations of the ϵ and δ parameters. The bottom row shows the correlation metrics for different models and combinations of the ϵ and δ parameters. Since the naive model always predicts the median value from the training set, the correlation metric is absent for this model.

The models demonstrated similar performance with the mean absolute error of ∼0.7 for random and homology splits with 0.2, 0.4, and 0.6 thresholds, respectively (see Table S10 and Figure 2). As for the Spearman’s correlation coefficient, the XGBoost and KNN models showed 0.59 and 0.58 values on the random split and a slight decrease to 0.50 and 0.49 values, respectively, for the homology split with the 0.6 threshold (see Table S10). It is important to note that many known enzymes work at close to neutral pH conditions. Indeed, for the enriched data set 5586 (56%) out of 10 031 sequences fall into the [6.0,8.0] pH range. As a consequence, a naive model that predicts 7.3 pH (the median value) for any protein sequence demonstrates a mean absolute error of ∼0.9. At the same time, one is typically more interested in detecting sequences with an optimal pH beyond the standard range. To avoid such a pitfall and verify the robustness of the derived models, we eliminated sequences with experimental pH values falling into δ = 0.5,1.0,1.5 vicinity of the median pH value of the data set (pH = 7.3) and recalculated the performance metrics (see Figure 2 and Table S10). We observed a gradual decrease in terms of the mean absolute error from 0.7 to 1.4 for the random split and homology splits, respectively, as δ increased from 0.0 to 1.5. While the mean absolute error increases as δ increases, we observed that the Spearman’s correlation coefficient does not change or even slightly improves (see Figure 2). Therefore, the OphPred model can be useful for protein screening campaigns, where one is typically interested in selecting Top N protein sequences for experimental validation.

#### Impact of Different Embeddings

The derived OphPred model is based on ESM-2, which is suitable to compute rich embeddings of protein sequences in high-throughput mode. However, there are other methods to calculate protein embeddings that can be also used to derive machine learning models for the downstream tasks. To test the impact of different embeddings for the optimal pH prediction problem, we considered one-hot encoding and deep learning-based embeddings from 11 language models with diverse architectures (CNN, RNN, LSTM, Transformers) and trained on different large databases (see Methods). We trained models using these embeddings and a homology split with a threshold value (ε) of 0.6. As expected, the transformer-based models (various modifications of ProtTrans and ESM) showed comparable performance, while CNN-, LSTM-, and RNN-based models performed slightly worse, and the one-hot encoding-based models demonstrated the worst performance (see Figure S6).

It is worth noting that databases used to train protein language models are typically biased with respect to the superkingdoms. For instance, there are twice as many bacterial sequences as eukaryotic sequences in the UniProt database. It may lead to an uneven distribution of species of sequences in the training set of large language models like ESM-2. To test whether the derived OphPred models are biased to the types of organisms, we retrieved information about the superkingdoms of the species from which protein sequences came and saw how the performance metrics are distributed across the superkingdoms. We observed that models’ metrics are similar for different superkingdoms on different splits; the largest discrepancy of ∼0.10 in the performance metric with respect to the entire test set was observed for the Archaea superkingdom subset (see Tables S12 and S13 and Figure S7). The fact that the metrics are not strongly influenced by the origin of the protein sequences indicates the applicability of the model to sequences from different superkingdoms.

#### Comparison with EpHod

We compared OphPred with EpHod, another sequence-based method for predicting enzyme optimal pH.^25^ For rigorous comparison, we retrieved the training (7,124 sequences) and test (1,972 sequences) subsets from the corresponding Zenodo repository (https://zenodo.org/records/8011249) and retrained our models from scratch. We observed that OphPred outperforms EpHod on the test set in terms of the mean absolute error, demonstrating a mean absolute error of 0.6 and a correlation coefficient of 0.55, while EpHod achieves a mean absolute error of 0.7 and a correlation of 0.59.

### PFAM and EC Splits

**Figure 3:**
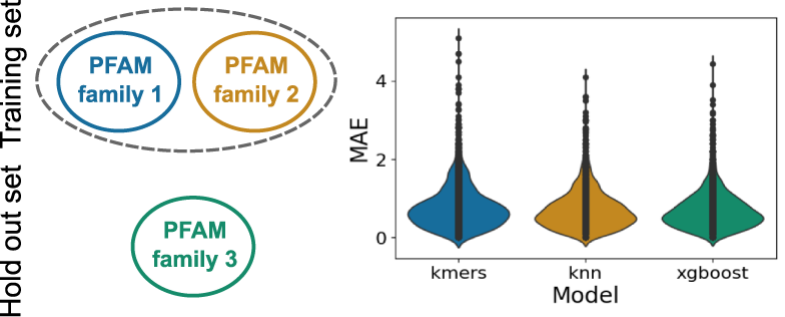
Left side of the figure schematically illustrates the PFAM-based split. The right side of the figure demonstrates the distribution of the MAE performance metric on the hold-out PFAM families for different models.

**Figure 4:**
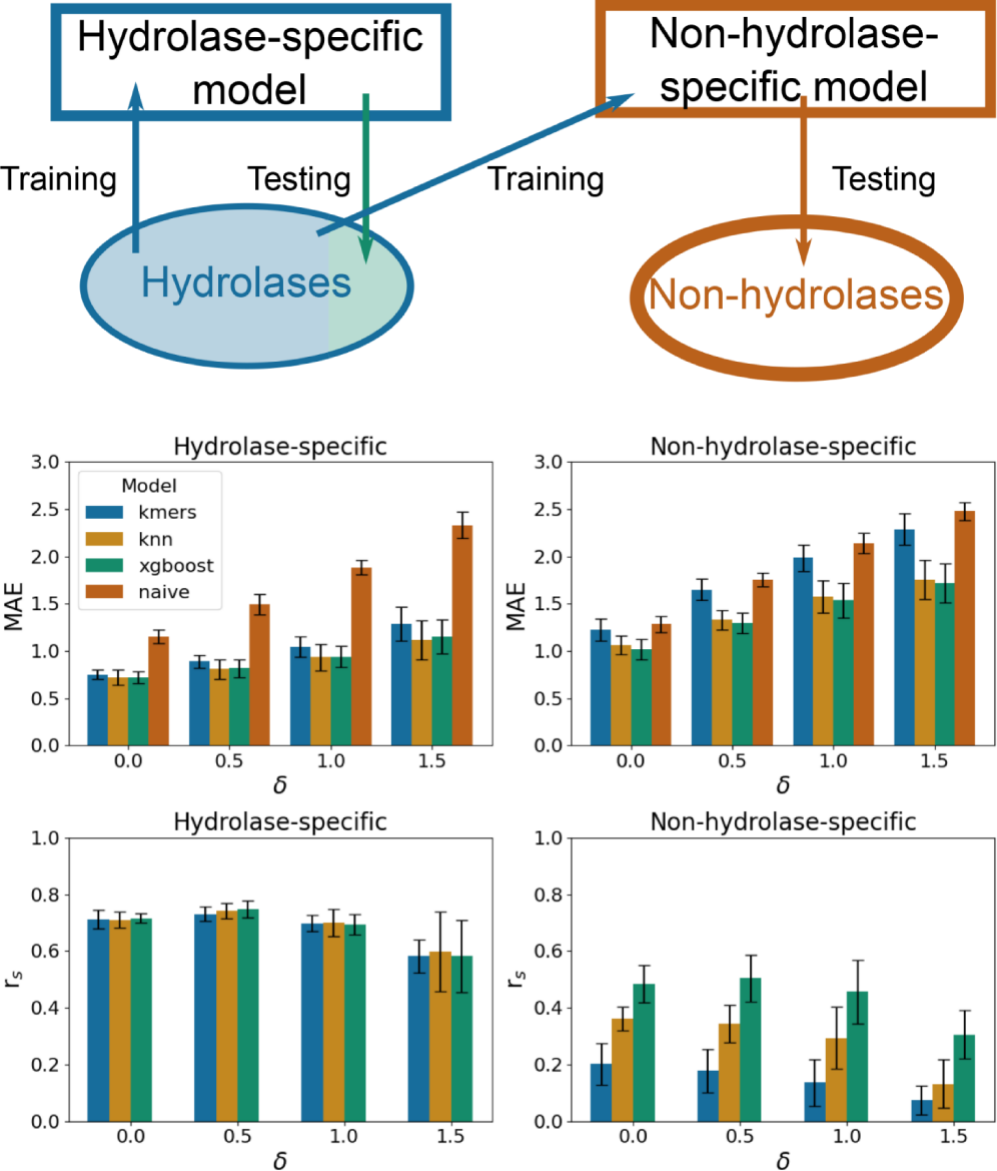
Performances of the hydrolase-specific and nonhydrolase-specific models. The top row schematically shows how both models were trained and tested. The middle row shows the MAE metrics for the hydrolase-specific (left) and the nonhydrolase-specific (right) models. The bottom row shows the correlation metric for the hydrolase-specific (left) and the nonhydrolase-specific (right) models. Since the naive model always predicts the median value from the training set, the correlation metric is absent for this model.

To diversify the train-test split strategy further, we carried out a hold-out evaluation based on the PFAM annotations (see Methods). We considered only mean absolute errors as the performance metrics because we observed a lot of small clusters corresponding to the same PFAM annotation (typically ≤10), hence nonrepresentative correlation coefficients. We observed similar performance in terms of the average mean absolute errors for the hold-out subsets (∼0.9), indicating the absence of apparent biases of the developed models with respect to particular protein families (see Figure 3). However, we also observed a larger std. value (∼±0.4), and Figure S1 shows the MAE along with the std. value with respect to the size of the cluster. Next, we carried out the EC-based split, where we trained the hydrolase-specific and nonhydrolase-specific models, and tested both models on the hydrolase sequences (see Methods). We found that the models performed better when trained on the same class of enzyme. For instance, OphPred-KNN trained on hydrolases achieves a mean absolute error of 0.7 ± 0.1 and a correlation of 0.71 ± 0.03, while the nonhydrolase-specific model demonstrated a mean absolute error of 1.1 ± 0.1 and a correlation of 0.36 ± 0.04. (see Figure 4) On the one hand, these results indicate a limitation of the derived models’ application to the novel protein classes; and on the other hand, it indicates the usability of the family specific models.

### OphPred Improves with New Data Available

**Figure 5:**
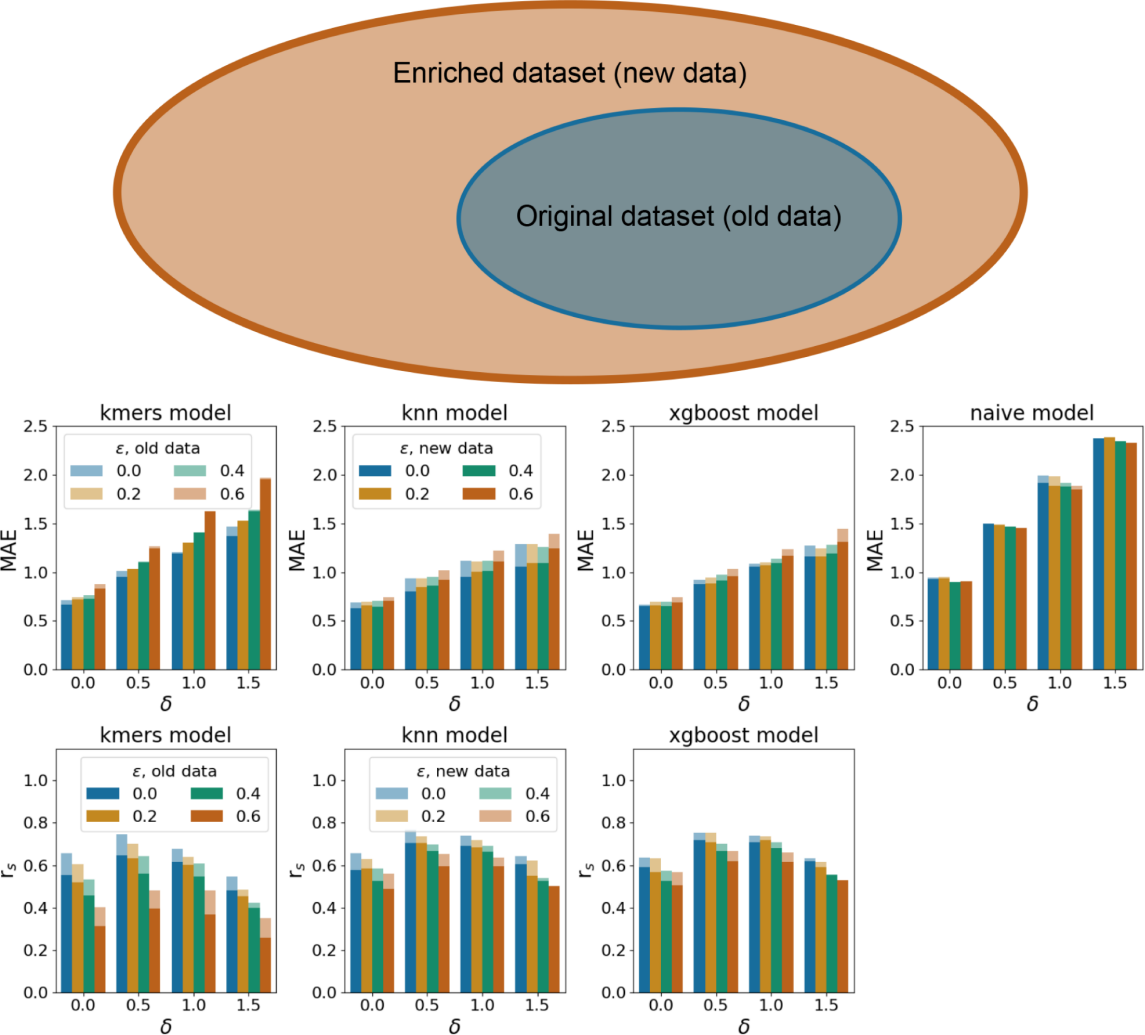
Effect of data enrichment on the performance of the models. The top row schematically shows the data enrichment. The middle row shows the MAE metrics for different models, where the metrics obtained with the original data set are shown with transparent bars, while metrics obtained with the enriched data set are shown with opaque bars. The bottom row shows the correlation metrics for different models, where the metrics obtained with the original data set are shown with opaque bars, while metrics obtained with the enriched data set are shown with transparent bars. Since the naive model always predicts the median value from the training set, the correlation metric is absent for this model.

With the rapid accumulation of new biophysical data, it is important for machine learning approaches to demonstrate improved performance over time. We observed that the OphPred models derived using the enriched training sets demonstrate ∼10% increase of the Spearman correlation coefficient, while approximately the same mean absolute errors compared to the nonenriched models (see Figure 5 and S3–5, Tables S8 and S10 for more details). Importantly, for a fair comparison, we kept the test set unchanged and enriched only the training sets (from ∼2,000 to ∼10,000 sequences). Thus, the OphPred approach can be enhanced to achieve even higher accuracy and predictive power with the accumulation of new optimal pH data.

To further explore the data set expansion, we considered the mean growth pH data. It has been shown for at least five different enzymes that the average temperature of the catalytic optimum correlates with the growth temperature of the organism.^28^ Furthermore, including information about the mean growth temperature of microorganisms improves the accuracy of the predictive models for the catalytic temperature of enzymes.^29^ Therefore, one may hypothesize that similarly, including information about the optimal growth pH should improve models for optimal enzyme pH prediction. To test if optimal and mean growth pH values are indeed related, we trained an additional model on the optimal growth pH data set (see Methods), which contains ∼50 times more sequences, compared to our optimal pH data set.

The derived model showed promising results on the validation sets corresponding to the mean growth pH values (the mean absolute errors ∼0.5–0.8 and Spearman’s correlation coefficients ∼0.65–0.77; see Table S10). However, it demonstrated poor results for the optimal enzyme pH data set. More specifically, we observed the mean absolute errors of ≥1.4 for optimal pH data sets and no correlation in terms of Spearman’s rank correlation coefficients. Thus, the obtained results indicate that the mean growth pH and the optimal enzyme pH are not strongly correlated. Additionally, we found 364 protein sequences that have both the mean growth pH and the optimal enzyme pH measured. The Pearson’s correlation coefficient between them is −0.17, which confirms the lack of strong relationships between mean growth pH and optimal enzyme pH. Nonetheless, the obtained results also show that the proposed approach is not limited to optimal enzyme pH prediction problem and can be used to derive target-specific predictive models.

## Conclusion

In this study, we have developed a machine learning-based approach, OphPred, to predict enzyme optimal pH. We considered different splitting strategies, including random, homology-based, EC-based, and PFAM-based splits, to test OphPred predictive power and observed a solid performance in terms of the mean absolute error and Spearman correlation coefficient. Additionally, we observed that OphPred benefits from adding new data to the training. OphPred operates with the protein sequence information only and, hence, is fast to screen large-size protein libraries. OphPred is available at https://github.com/i-Molecule/optimalPh. and https://research.constructor.tech/public/project/optimalph.

## Methods

### Data Sets

#### Optimal pH

We retrieved entries from the two versions of the Brenda-Enzymes database^26^ (version November 2021 and version March 2023) with known optimal pH values, as well as the optimal pH range. For the latter case, we assigned the optimal pH value to an entry as the average of the lower and upper boundaries of the optimal pH range. Note that the database does not contain the protein sequences, but the protein name, EC number,^30^ organism information, and, in rare cases, the UniProt accession identifier. To avoid data ambiguity, we considered only entries with UniProt accession identifiers and retrieved the corresponding protein sequences. For the sequences with several pH values, we calculated the standard deviation (std.) of the pH values and discarded sequences with std. > 1.0; for the remaining sequences we used the averaged pH value, as the optimal enzyme pH. In total, we obtained two data sets consisting of 2,840 (for Brenda-Enzymes database version November 2021) and 10,031 (for Brenda-Enzymes database version March 2023) protein sequences with assigned optimal pH values. Additionally, we extracted taxonomy information from the Uniprot accession identifiers: 5,020 proteins belong to eukaryotes, 4,090 to bacteria, 724 to archaea, 72 to viruses, and 125 remained unclassified.

#### Mean Growth pH

In addition, we collected 2,516,572 protein sequences with known mean growth pH values from the GOLD database.^31^ Note that the same proteins may (i) occur in different organisms and (ii) correspond to several measurements; therefore, each sequence may be associated with different growth pH values. Indeed, we observed only 252,491 unique protein sequences. For consistency, we discarded sequences associated with multiple growth pH values, if the corresponding standard deviation is larger than 1.0, resulting in 169,517 protein sequences. As the mean growth pH, we used the averaged value according to
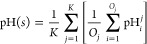
1where *K* is the number of different organisms with known mean growth pH for the protein sequence *s*, *O*_*j*_ is the total number of measurements for *s* within the organism *j*, and  is the corresponding measurement.

### Train and Validation Splits

Train and validation splitting are vital parts of computational experiments. Therefore, to evaluate the robustness of the developed approach, we divided the data sets into training and validation parts using four different splitting strategies: (i) the random split, (ii) the homology split, (iii) the PFAM split, and (iv) the EC split, as follows.

#### Random Split

For the random split, we simply divided data sets randomly into the train and validation subsets with a 3:1 ratio. Note that commonly used random split likely leads to overestimated results due to the highly similar protein sequences shared between the training and validation subsets.

#### Homology Split

To overcome potential bias related to the random split, one can group sequences based on their sequence similarity followed by the cluster-based split, such that any two similar sequences together belong either to train or validation set. For each data set, we constructed multiple sequence alignments using MAFFT(v7.450)^32^ with default parameters except for the option “−anysymbol” which was turned on to guarantee the correctness of parsing of sequences with noncanonical amino acids. Then we calculated pairwise sequence similarity matrix |*S*|, where the pairwise scores were divided by the length of the shortest sequence providing a more strict clusterization criterion. Next, we calculated the distance matrix as |*I*|−|*S*|, where |*I*| is the matrix of ones. Then, we used the DBSCAN^33^ algorithm implemented in the sklearn python library^34^ to obtain clusters from the distance matrix. Thus, any pair of sequences from two different clusters has distance ≤ ε, where ε is the input threshold. We tested three different values of the threshold parameter ε: 0.2, 0.4, and 0.6. Note that this procedure may result in orphan sequences, i.e., not assigned to any cluster; in such cases, we grouped all the orphan sequences into a separate cluster. For example, for the optimal pH data set using *ε* = 0.2 leads to the orphan cluster comprising 85% of the Brenda-Enzymes data set (version November 2021) (see Table S1). Finally, the obtained clusters were split in a way to preserve the 3:1 ratio with respect to the number of sequences between the training and validation sets. Table S1 lists clusterization details for the mean growth pH and enzyme optimal pH data sets. Note that rigorous clusterization of the mean growth pH data set is not feasible, as it requires dozens of billions of comparisons and ∼100 GB RAM for DBSCAN. Instead, we used CD-HIT^35^ for homology-based clusterization of the mean growth pH data set. CD-HIT is suitable for large sets of sequences, although it does not guarantee the absence of similar sequences between two different clusters. We set the ε parameter to 0.6 for clusterization with CD-HIT because smaller values lead to degradation in both speed and accuracy of clusterization.^36^

#### PFAM Split

The PFAM database of protein families^37^ annotates each entry with one of six different types: family, domain, motif, repeat, coiled-coil, or disordered, indicating the class of the functional unit being represented by that entry. Given a protein sequence, one can retrieve the PFAM annotation by searching against the PFAM library of Hidden Markov Model profiles calculated from the PFAM’s MSAs. We obtained the PFAM annotations for 2,774 out of 2,840 sequences from the Brenda-Enzymes data set (version November 2021), 9,784 out of 10 031 from the Brenda-Enzymes data set (version March 2023), as well as for 165,820 out of 169,517 sequences from the mean growth pH data set, using the PfamScan web service Python client as of December 2023 https://github.com/ebi-wp/webservice-clients-generator. It is important to note that each sequence generally corresponds to several PFAM numbers. Then we used the hold-out validation, where given a PFAM number, all sequences corresponding to this number are assigned to the validation set and the remaining sequences are assigned to the train set. In total, we composed 1,363, 2,556, and 1,403 holdout splits for two Brenda-Enzymes data sets and the mean growth pH data set, respectively.

#### EC Split

All enzymes are classified based on the chemical reactions they catalyze using a four-number code, that is the EC (Enzyme Commission) code.^30^ For example, coniferyl alcohol dehydrogenase has a 1.1.1.194 EC code, where the first number shows that the protein belongs to one of the following classes: Oxidoreductases (1), Transferases (2), Hydrolases (3), Lyases (4), Isomerases (5), Ligases (6), and Translocases (7), and the other three numbers reflect the classification into smaller and more specific subclasses. For the optimal enzyme pH data sets, we retrieved the corresponding EC numbers from the Brenda-Enzymes database. As for the mean growth pH data set, we used Swiss-Prot^38^ and ECDomainMiner^39^ to classify protein sequences into seven groups corresponding to the top-level EC numbers, and we discarded sequences with unknown EC numbers. We observed that most of the sequences belong to the non-Hydrolase family; therefore, we put all such sequences in the training set and Hydrolase sequences in the validation set. Note that we discarded proteins with both hydrolase and nonhydrolase functions from consideration. In total, we obtained 1,165 hydrolase sequences with known optimal enzyme pH. Next, we trained the hydrolase-specific and nonhydrolase-specific models as follows. First, we prepared five folds from 1,165 of hydrolase sequences. For the hydrolase-specific model, we used these folds for the cross-validation. As for the nonhydrolase-specific models, we used each fold as a validation fold, while taking 1,675 nonhydrolase sequences as the training set. Note that in contrast to the PFAM-based and homology-based splits, the EC-based split does not rely on the sequence or structural information on a protein. Indeed, proteins with different 3D structures can catalyze the same reaction and have the same EC number; for example, human and bovine peptidyl-proline cis–trans isomerases have different folds (PDB IDs: 1PIN and 1IHG). Noteworthy, one protein may catalyze different types of chemical reactions, for example, the folD protein from *E. coli* (Uniprot ID: P24186) is bifunctional and acts as both hydrolase and oxidoreductase. Therefore, the EC-based split represents a complementary way to split protein sequences.

### Enrichment

To demonstrate the potential of OphPred to improve with the accumulation of new biophysical data, we extended the training set using a newer version of the Brenda-Enzymes data set (version March 2023). For the random split, we simply added new sequences to the training set, and for the homology split, we added new sequences considering the homology with respect to the test set to ensure the absence of similar sequences between the training and test sets. For the EC split, we enriched the training set with the nonhydrolase sequences for the nonhydrolase-specific model, while the test set consisting of the hydrolase sequences was the same; and for the hydrolase-specific model, we performed 5-fold cross-validation using the enriched training set. As for the PFAM split, we used the same strategy to compose the hold-out splits as for the Brenda-Enzymes data set (version November 2021).

### The Baseline Approach

As the baseline, we used a two-step approach, that outperformed all the methods in Novozymes (https://www.novozymes.com/en) challenge of Predict optimal pH for enzyme activity https://biohackathon.biolib.com/event/2021-protein-edition. The first step is to calculate the property values of short sequence fragments, and the second step is to predict the property value of the entire sequence from the property values of its fragments. More precisely, we represent each protein sequence in the training set as a set of *k*-mers, which are subsequences of length *k*, and associate each *k*-mer with the pH value of the protein sequence. Generally, a particular *k*-mer can have more than one associated pH value because it can be observed in more than one sequence. Thus, each *k*-mer corresponds to the list of pH values, so we calculated the mean, maximum, and minimum pH values for it. Note that the first step is done only once for the given training set.

To predict optimal pH of the input protein sequence on the second step let us denote T a 20^*k*^-vector of the pH values of all *k*-mers listed in the lexicographical order, and assign *T*_i_ = 0, if the corresponding *k*-mer is absent in the *k*-mer table. Let us also denote P as a 20^*k*^-vector for a given protein sequence, where *P*_*i*_ is the number of occurrences of the *i*-th k-mer in the protein. Then we calculate the optimal pH of the protein sequence as
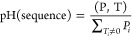
2

In practice T is sparse (the number of nonzero elements is ≪20^*k*^); therefore, it is much more efficient to directly iterate over the vocabulary of *k*-mers and calculate the pH value as
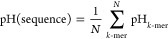
3where *N* is the number of *k*-mers in a protein sequence. Similarly, one can estimate the optimal pH range for an enzyme by calculating the minimal and maximal pH value as the lower and upper bound of the pH range, respectively.

### The OphPred Approach

To encode protein sequences as numerical vectors we used the Evolutionary Scale Model (ESM-2) which is one of the largest transformer-like language models specifically trained for protein sequences—it comprised 33 neural network layers and 650 million trainable parameters.^40^ Note that we cut 13 protein sequences to the first 5000 amino acids due to the limitations of the GPU memory (NVIDIA GeForce GTX 1080 Ti 12GB). The ESM-2 model takes protein sequence as the input and yields a 1280-size vector in the output, reflecting the structural and functional properties of the protein. We used a pretrained ESM-2 model from the fair-esm (v 2.0.0 as of December 2023) python package https://github.com/facebookresearch/esm.^40^ Given the numerical representations of the protein sequences, we then used k-nearest neighbor (KNN) and XGBoost as the regression models for the optimal pH prediction tasks. We determined the optimal parameters of the regressors using the grid search (Tables S2 and S3). Therefore, we obtained end-to-end models, named the OphPred models, which take a protein sequence as the input and output its optimal pH value.

### Embeddings

With the advances in deep learning language models, it becomes possible to efficiently represent protein sequences as high-dimensional vectors or embeddings. While we mainly focused on the ESM-2 model to obtain the embeddings, we also considered the following methods for comparison:

- Averaged one-hot encoded vectors over amino acid positions in a protein sequence.
- A Recurrent Neural Network (RNN) model by Bepler,^41^ which was trained on ∼21*M* sequences from the PFAM database. The model is a stack of 3 bidirectional Long Short-Term Memory (LSTM) layers followed by a linear layer, that gives embeddings for each position. The embeddings are then averaged to obtain the protein embedding.
- The CPCPProt model,^42^ which was trained on ∼32*M* sequences from the PFAM database.^37^ The model comprises a 1d-Convolutional Neural Network that converts sequence patches of length 11 into numerical vectors fed into a Recurrent Neural Network model to obtain the position embeddings. The embeddings are then averaged to obtain the protein embedding.
- The RNN-based SeqVec model,^43^ trained on the UniRef50 data set (∼45*M* sequences).^38^ The model consists of two stacked bidirectional LSTM layers, that output embeddings for each position. The position embeddings are then averaged to obtain the protein embedding.
- The RNN-based PLUS model,^44^ which was trained on ∼14*M* sequences from the PFAM database.^37^ The model is a stack of 3 bidirectional RNNs followed by a linear layer, that gives position-wise embeddings. The embeddings are then averaged to obtain the protein embedding.
- The transformer-based ProtTrans_BERT/Albert/T5 models.^45^ We considered three variants of ProtTrans_T5 models, which were trained on sequences from BFD (https://bfd.mmseqscom.), UniRef50,^38^ or both databases. Models ProtTrans_BERT and ProtTrans_Albert were trained on protein sequences from the BFD database. The models comprise different variations of the stacked transformer blocks to obtain position-wise embeddings, which are then averaged, resulting in protein embedding.
- The transformer-based ESM-1 and ESM-1b^46^ models, trained on the UniRef50 data set.^38^ The models consist of 33 and 34 transformer blocks, respectively. Similarly, the models’ output is position embeddings, which are averaged to obtain the protein embedding.

To compute embeddings for the ESM family models, we used the fair-esm python package (https://github.com/facebookresearch/esm, v 2.0.0 as of December 2023). For the other embeddings, we used the bio_embeddings (v 0.2.2) python package.^47^

### Performance Metrics

To assess the performance of the methods, we used Spearman’s Rank Correlation Coefficient and Mean Absolute Error:
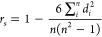
4
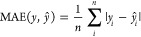
5where *d*_*i*_ is the difference between the true and the predicted ranks for sample *i* and *n* is the total number of samples.

## Data Availability

Additional details are available in the Supporting Information. OphPred is available at https://github.com/i-Molecule/optimalPh. and https://research.constructor.tech/public/project/optimalph.

## Supporting Information Available

Grid search details; information about clustering for homology splitting; tables with the performance metric values; plots of the performance metric values for different models (PDF)
