# Supplementary material for: Approaching Optimal pH Enzyme Prediction with Large Language Models
Source: ACS Synth Biol. 2024 Aug 28;13(9):3013–21. doi: 10.1021/acssynbio.4c00465 (PMC11421216; doi:10.1021/acssynbio.4c00465)
Supplement: Supplementary file 1 — sb4c00465_si_001.pdf [file sb4c00465_si_001.pdf]

Supplement Information for: Approaching Optimal pH Enzyme Prediction with Large Language Models

Mark Zaretckii,[†,‡] Pavel Buslaev,[¶] Igor Kozlovskii,[†,‡] Alexander Morozov,[§] and Petr Popov[*,†,‡,‖]

† *Tetra D AG, 8200 Shaffhausen, Switzerland*

‡ *Constructor University Bremen gGmbH, 28759 Bremen, Germany*

¶ *Nanoscience Center and Department of Chemistry, University of Jyväskylä, 40014, Jyväskylä, Finland*

§ *Independent researcher*

‖ *Constructor Technology AG, 8200 Shaffhausen, Switzerland*

E-mail: ppopov@constructor.university

Tables

Table S1: Information about clusters.(Clusters was prepared with DBSCAN and CD-HIT for Brenda and Mean Growth pH datasets, respectively.)

	Seqs	Threshold	Orphan seqs	Min cluster size	Max cluster size	Mean cluster size	Median cluster size
	2840	0.2	2420	2	10	5.0	4.5
Brenda(Version March, 2021)	2840	0.4	1984	2	15	7.0	6.5
	2840	0.6	1329	2	21	10.4	10.0
Mean Growth pH	169517	0.6	17476	2	2278	277.4	173.0

Table S2: Information about parameters probed during grid search for XGBoost

Hyperparameter	Tested	Optimal
colsample_bytree	[0.5, 0.8]	0.8
learning rate	[0.05, 0.20, 0.30]	0.2
n_estimators	[10, 20, 30]	30
max_depth	[3, 6, 10]	6
reg_alpha	[0, 1, 5]	1.0
reg_lambda	[0, 1, 5]	0.0
min_child_weight	[1, 3, 7, 16]	7
gamma	[0.0, 0.2, 0.4]	0.4

Table S3: Information about parameters probed during grid search for kNN

Hyperparameter	Tested	Optimal
n_neighbours	[3, 5, 7, 10]	7

Table S4: The performance metrics observed for various methods with amino acid frequencies as a feature vectors, δ values (distance from $pH = 7.3$) and ϵ values (homology threshold, 0.0 stands for random split) for Brenda-Enzymes with UniProt identifiers.

		δ							
		0.0		0.5		1.0		1.5	
ϵ	model	mae	correlation	mae	correlation	mae	correlation	mae	correlation
0.0	knn	0.8 ± 0.0	0.47 ± 0.02	1.0 ± 0.0	0.59 ± 0.03	1.3 ± 0.1	0.62 ± 0.02	1.5 ± 0.1	0.54 ± 0.03
	xgboost	0.8 ± 0.0	0.46 ± 0.02	1.1 ± 0.0	0.58 ± 0.03	1.3 ± 0.1	0.61 ± 0.02	1.6 ± 0.1	0.54 ± 0.04
0.2	knn	0.8 ± 0.0	0.45 ± 0.03	1.1 ± 0.1	0.56 ± 0.04	1.4 ± 0.1	0.56 ± 0.05	1.6 ± 0.1	0.45 ± 0.08
	xgboost	0.8 ± 0.0	0.45 ± 0.03	1.1 ± 0.0	0.55 ± 0.04	1.4 ± 0.1	0.57 ± 0.05	1.6 ± 0.1	0.44 ± 0.08
0.4	knn	0.8 ± 0.0	0.4 ± 0.01	1.1 ± 0.0	0.51 ± 0.02	1.4 ± 0.0	0.52 ± 0.06	1.6 ± 0.1	0.43 ± 0.06
	xgboost	0.8 ± 0.0	0.4 ± 0.02	1.1 ± 0.0	0.5 ± 0.02	1.4 ± 0.0	0.53 ± 0.05	1.6 ± 0.1	0.44 ± 0.04
0.6	knn	0.8 ± 0.0	0.42 ± 0.02	1.1 ± 0.0	0.52 ± 0.03	1.4 ± 0.1	0.53 ± 0.06	1.7 ± 0.1	0.46 ± 0.09
	xgboost	0.8 ± 0.0	0.44 ± 0.01	1.1 ± 0.0	0.53 ± 0.01	1.4 ± 0.0	0.54 ± 0.06	1.6 ± 0.1	0.49 ± 0.07
ec	knn	1.1	0.22	1.1	0.22	1.1	0.22	1.1	0.22
	xgboost	1.1	0.25	1.1	0.25	1.1	0.25	1.1	0.25

Table S5: The performance metrics observed for various methods with dipeptide frequencies as a feature vectors, δ values (distance from $pH = 7.3$) and ϵ values (homology threshold, 0.0 stands for random split) for Brenda-Enzymes with UniProt identifiers.

		δ							
		0.0		0.5		1.0		1.5	
ϵ	model	mae	correlation	mae	correlation	mae	correlation	mae	correlation
0.0	knn	0.8 ± 0.0	0.35 ± 0.04	1.2 ± 0.0	0.48 ± 0.05	1.5 ± 0.1	0.52 ± 0.06	1.8 ± 0.1	0.48 ± 0.06
	xgboost	0.8 ± 0.0	0.45 ± 0.02	1.1 ± 0.0	0.57 ± 0.02	1.4 ± 0.0	0.59 ± 0.02	1.6 ± 0.1	0.49 ± 0.06
0.2	knn	0.8 ± 0.0	0.36 ± 0.04	1.2 ± 0.0	0.46 ± 0.04	1.5 ± 0.0	0.47 ± 0.03	1.8 ± 0.1	0.4 ± 0.01
	xgboost	0.8 ± 0.0	0.41 ± 0.03	1.2 ± 0.0	0.53 ± 0.04	1.4 ± 0.0	0.53 ± 0.05	1.7 ± 0.1	0.4 ± 0.1
0.4	knn	0.8 ± 0.0	0.27 ± 0.02	1.2 ± 0.0	0.38 ± 0.03	1.6 ± 0.0	0.42 ± 0.03	1.8 ± 0.1	0.39 ± 0.03
	xgboost	0.8 ± 0.0	0.37 ± 0.03	1.2 ± 0.0	0.47 ± 0.05	1.5 ± 0.0	0.49 ± 0.07	1.7 ± 0.1	0.4 ± 0.08
0.6	knn	0.8 ± 0.0	0.28 ± 0.02	1.2 ± 0.0	0.37 ± 0.03	1.6 ± 0.0	0.37 ± 0.07	1.9 ± 0.1	0.34 ± 0.11
	xgboost	0.8 ± 0.0	0.35 ± 0.01	1.2 ± 0.0	0.44 ± 0.02	1.5 ± 0.0	0.46 ± 0.06	1.8 ± 0.1	0.4 ± 0.09
ec	knn	1.1	0.23	1.1	0.23	1.1	0.23	1.1	0.23
	xgboost	1.1	0.22	1.1	0.22	1.1	0.22	1.1	0.22

Table S6: The performance metrics observed for various methods with amino acid frequencies as feature vectors and δ values (distance from $pH = 6.0$) and ϵ values (homology threshold, 0.0 stands for random split) for mean growth pH dataset. EC stands for hydrolases/non-hydrolases split.

		δ							
		0.0		0.5		1.0		1.5	
ϵ	model	mae	correlation	mae	correlation	mae	correlation	mae	correlation
0.0	knn	0.6 ± 0.0	0.7 ± 0.0	0.6 ± 0.0	0.71 ± 0.0	0.8 ± 0.0	0.72 ± 0.0	0.9 ± 0.0	0.53 ± 0.01
	xgboost	1.0 ± 0.0	0.38 ± 0.0	1.2 ± 0.0	0.4 ± 0.0	1.4 ± 0.0	0.43 ± 0.0	1.7 ± 0.0	0.41 ± 0.0
0.6	knn	1.0 ± 0.0	0.25 ± 0.02	1.2 ± 0.0	0.28 ± 0.02	1.3 ± 0.0	0.31 ± 0.02	1.6 ± 0.0	0.32 ± 0.03
	xgboost	1.0 ± 0.0	0.25 ± 0.03	1.2 ± 0.0	0.28 ± 0.03	1.4 ± 0.0	0.32 ± 0.02	1.8 ± 0.0	0.36 ± 0.01
ec	knn	1.1	0.13	1.1	0.13	1.1	0.13	1.1	0.13
	xgboost	1.1	0.2	1.1	0.2	1.1	0.2	1.1	0.2

Table S7: The performance metrics observed for various methods with amino acid frequencies as feature vectors and δ values (distance from $pH = 6.0$) and ϵ values (homology threshold, 0.0 stands for random split) for mean growth pH dataset. EC stands for hydrolases/non-hydrolases split.

		δ							
		0.0		0.5		1.0		1.5	
ϵ	model	mae	correlation	mae	correlation	mae	correlation	mae	correlation
0.0	knn	0.6 ± 0.0	0.71 ± 0.0	0.6 ± 0.0	0.71 ± 0.0	0.8 ± 0.0	0.72 ± 0.0	0.9 ± 0.0	0.54 ± 0.01
	xgboost	1.0 ± 0.0	0.43 ± 0.0	1.1 ± 0.0	0.45 ± 0.0	1.3 ± 0.0	0.47 ± 0.0	1.6 ± 0.0	0.42 ± 0.01
0.6	knn	1.1 ± 0.0	0.22 ± 0.01	1.2 ± 0.0	0.24 ± 0.01	1.4 ± 0.0	0.29 ± 0.01	1.7 ± 0.0	0.29 ± 0.03
	xgboost	1.1 ± 0.0	0.25 ± 0.03	1.2 ± 0.0	0.28 ± 0.03	1.4 ± 0.0	0.32 ± 0.03	1.8 ± 0.0	0.34 ± 0.02
ec	knn	1.2	-0.01	1.2	-0.01	1.2	-0.01	1.2	-0.01
	xgboost	1.1	0.13	1.1	0.13	1.1	0.13	1.1	0.13

Table S8: The performance metrics observed for various methods on hydrolases vs. non-hydrolases split and δ values (distance from $pH = 7.3$).

		δ						
		0.0		0.5		1.0		
		mae	correlation	mae	correlation	mae	correlation	mae
Method	Strategy							
kmers	enriching	1.2 ± 0.1	0.2 ± 0.06	1.6 ± 0.1	0.18 ± 0.07	2.0 ± 0.1	0.14 ± 0.07	2.3 ± 0.2
	simple	1.2 ± 0.1	0.14 ± 0.1	1.6 ± 0.1	0.15 ± 0.11	1.9 ± 0.1	0.11 ± 0.12	2.2 ± 0.1
knn	enriching	1.1 ± 0.1	0.36 ± 0.04	1.3 ± 0.1	0.34 ± 0.06	1.6 ± 0.2	0.3 ± 0.1	1.8 ± 0.2
	simple	1.1 ± 0.1	0.35 ± 0.06	1.4 ± 0.1	0.37 ± 0.07	1.6 ± 0.1	0.3 ± 0.07	1.8 ± 0.1
xgboost	enriching	1.0 ± 0.1	0.47 ± 0.05	1.3 ± 0.1	0.49 ± 0.03	1.5 ± 0.2	0.46 ± 0.09	1.7 ± 0.2
	simple	1.1 ± 0.1	0.33 ± 0.05	1.4 ± 0.1	0.35 ± 0.03	1.7 ± 0.1	0.35 ± 0.07	1.9 ± 0.1

Table S9: The performance metrics observed for various methods, δ values (distance from $pH = 6.0$) and ϵ values (homology threshold, 0.0 stands for random split) for mean growth pH dataset. Column split indicates whether train set has been enriched with more recent data(see Enrichment subsection on Methods) or not. EC stands for hydrolases/non-hydrolases split.

		δ							
		0.0		0.5		1.0		1.5	
ϵ	model	mae	correlation	mae	correlation	mae	correlation	mae	correlation
0.0	baseline	0.6 ± 0.0	0.81 ± 0.0	0.6 ± 0.0	0.81 ± 0.0	0.8 ± 0.0	0.82 ± 0.01	1.0 ± 0.0	0.68 ± 0.01
	knn	0.5 ± 0.0	0.77 ± 0.0	0.5 ± 0.0	0.78 ± 0.0	0.6 ± 0.0	0.79 ± 0.01	0.7 ± 0.0	0.63 ± 0.01
	xgboost	0.8 ± 0.0	0.65 ± 0.01	0.9 ± 0.0	0.68 ± 0.01	1.0 ± 0.0	0.71 ± 0.01	1.2 ± 0.0	0.61 ± 0.01
0.6	baseline	1.0 ± 0.0	0.3 ± 0.03	1.2 ± 0.0	0.33 ± 0.03	1.4 ± 0.0	0.38 ± 0.03	1.9 ± 0.0	0.37 ± 0.03
	knn	1.0 ± 0.0	0.32 ± 0.02	1.1 ± 0.0	0.36 ± 0.02	1.2 ± 0.0	0.43 ± 0.01	1.4 ± 0.0	0.44 ± 0.02
	xgboost	1.0 ± 0.0	0.4 ± 0.03	1.1 ± 0.0	0.45 ± 0.02	1.2 ± 0.0	0.51 ± 0.01	1.5 ± 0.0	0.51 ± 0.01
ec	baseline	1.1	0.03	1.3	0.03	1.6	0.03	2.2	0.04
	knn	1.0	0.27	1.0	0.27	1.0	0.27	1.0	0.27
	xgboost	1.1	0.22	1.1	0.22	1.1	0.22	1.1	0.22

Table S10: The performance metrics observed for various methods, δ values (distance from $pH = 7.3$) and ϵ values (homology threshold, 0.0 stands for random split) for Brenda-Enzymes with UniProt identifiers. Column split indicates whether train set has been enriched with more recent data(see Enrichment subsection on Methods) or not.

			δ							
			0.0		0.5		1.0		1.5	
			mae	correlation	mae	correlation	mae	correlation	mae	correlation
threshold	type	split								
0.0	kmers	enriching	0.7 ± 0.0	0.66 ± 0.02	1.0 ± 0.1	0.75 ± 0.02	1.2 ± 0.1	0.68 ± 0.02	1.4 ± 0.1	0.55 ± 0.05
		simple	0.7 ± 0.0	0.55 ± 0.04	1.0 ± 0.1	0.65 ± 0.03	1.2 ± 0.1	0.61 ± 0.03	1.5 ± 0.1	0.48 ± 0.06
	knn	enriching	0.6 ± 0.0	0.66 ± 0.02	0.8 ± 0.0	0.77 ± 0.02	1.0 ± 0.1	0.74 ± 0.02	1.1 ± 0.1	0.64 ± 0.05
		simple	0.7 ± 0.0	0.58 ± 0.02	0.9 ± 0.0	0.71 ± 0.02	1.1 ± 0.0	0.69 ± 0.02	1.3 ± 0.1	0.61 ± 0.07
	xgboost	enriching	0.7 ± 0.0	0.63 ± 0.02	0.9 ± 0.0	0.75 ± 0.01	1.1 ± 0.1	0.72 ± 0.01	1.2 ± 0.1	0.62 ± 0.06
		simple	0.7 ± 0.0	0.58 ± 0.03	0.9 ± 0.0	0.71 ± 0.03	1.1 ± 0.1	0.71 ± 0.03	1.3 ± 0.1	0.61 ± 0.03
0.2	kmers	enriching	0.7 ± 0.0	0.61 ± 0.02	1.0 ± 0.1	0.7 ± 0.02	1.3 ± 0.1	0.64 ± 0.02	1.5 ± 0.1	0.49 ± 0.05
		simple	0.7 ± 0.0	0.52 ± 0.02	1.0 ± 0.1	0.63 ± 0.02	1.2 ± 0.1	0.6 ± 0.03	1.5 ± 0.1	0.45 ± 0.06
	knn	enriching	0.7 ± 0.0	0.63 ± 0.02	0.8 ± 0.0	0.74 ± 0.03	1.0 ± 0.1	0.72 ± 0.02	1.1 ± 0.1	0.62 ± 0.06
		simple	0.7 ± 0.0	0.59 ± 0.03	0.9 ± 0.0	0.7 ± 0.03	1.1 ± 0.1	0.69 ± 0.03	1.3 ± 0.0	0.55 ± 0.04
	xgboost	enriching	0.7 ± 0.0	0.63 ± 0.02	0.9 ± 0.0	0.75 ± 0.02	1.1 ± 0.0	0.74 ± 0.03	1.2 ± 0.0	0.62 ± 0.05
		simple	0.7 ± 0.0	0.58 ± 0.01	1.0 ± 0.0	0.71 ± 0.02	1.1 ± 0.0	0.73 ± 0.02	1.3 ± 0.0	0.59 ± 0.04
0.4	kmers	enriching	0.7 ± 0.0	0.53 ± 0.01	1.1 ± 0.0	0.64 ± 0.01	1.4 ± 0.0	0.61 ± 0.03	1.6 ± 0.1	0.42 ± 0.02
		simple	0.8 ± 0.0	0.46 ± 0.02	1.1 ± 0.0	0.56 ± 0.03	1.4 ± 0.0	0.55 ± 0.04	1.6 ± 0.1	0.4 ± 0.03
	knn	enriching	0.6 ± 0.0	0.59 ± 0.02	0.9 ± 0.0	0.7 ± 0.01	1.0 ± 0.0	0.69 ± 0.04	1.1 ± 0.1	0.54 ± 0.05
		simple	0.7 ± 0.0	0.53 ± 0.03	1.0 ± 0.0	0.67 ± 0.02	1.1 ± 0.0	0.66 ± 0.04	1.3 ± 0.1	0.53 ± 0.04
	xgboost	enriching	0.7 ± 0.0	0.58 ± 0.02	0.9 ± 0.0	0.7 ± 0.02	1.1 ± 0.0	0.71 ± 0.05	1.2 ± 0.1	0.58 ± 0.07
		simple	0.7 ± 0.0	0.52 ± 0.02	1.0 ± 0.0	0.67 ± 0.03	1.1 ± 0.0	0.69 ± 0.05	1.3 ± 0.1	0.54 ± 0.04
0.6	kmers	enriching	0.8 ± 0.0	0.4 ± 0.03	1.2 ± 0.1	0.48 ± 0.03	1.6 ± 0.1	0.48 ± 0.03	2.0 ± 0.1	0.35 ± 0.07
		simple	0.9 ± 0.0	0.31 ± 0.04	1.3 ± 0.0	0.39 ± 0.03	1.6 ± 0.0	0.37 ± 0.02	2.0 ± 0.1	0.26 ± 0.1
	knn	enriching	0.7 ± 0.0	0.56 ± 0.03	0.9 ± 0.0	0.65 ± 0.03	1.1 ± 0.1	0.64 ± 0.03	1.2 ± 0.1	0.49 ± 0.07
		simple	0.7 ± 0.0	0.49 ± 0.04	1.0 ± 0.0	0.59 ± 0.04	1.2 ± 0.1	0.6 ± 0.04	1.4 ± 0.1	0.5 ± 0.07
	xgboost	enriching	0.7 ± 0.0	0.56 ± 0.04	1.0 ± 0.0	0.67 ± 0.05	1.2 ± 0.1	0.67 ± 0.06	1.3 ± 0.1	0.55 ± 0.07
		simple	0.7 ± 0.0	0.5 ± 0.04	1.0 ± 0.0	0.61 ± 0.05	1.2 ± 0.1	0.62 ± 0.03	1.5 ± 0.1	0.53 ± 0.08

Table S11: The performance metrics observed for cross-validation on hydrolases for various methods, values (distance from pH = 7.3).

	δ							
	0.0		0.5		1.0		1.5	
model	mae	correlation	mae	correlation	mae	correlation	mae	correlation
kmers	0.9 ± 0.0	0.57 ± 0.07	1.0 ± 0.0	0.6 ± 0.07	1.2 ± 0.0	0.59 ± 0.08	1.5 ± 0.1	0.55 ± 0.08
knn	0.8 ± 0.1	0.64 ± 0.03	0.9 ± 0.1	0.67 ± 0.03	1.1 ± 0.1	0.66 ± 0.04	1.4 ± 0.2	0.65 ± 0.06
xgboost	0.8 ± 0.1	0.63 ± 0.03	0.9 ± 0.1	0.66 ± 0.03	1.1 ± 0.1	0.65 ± 0.02	1.4 ± 0.1	0.61 ± 0.06

Table S12: Difference between the metrics for OphPred with XGBoost head calculated using the sequences from one super kingdom and the metrics calculated using all the sequences from the test set for the random($\varepsilon = 0.0$) and homology splits.

	metric	homology threshold(ε)	Bacteria (superkingdom)	Eukaryota (superkingdom)	Archaea (superkingdom)
0	mae	0.0	0.0 ± 0.0	0.1 ± 0.0	0.1 ± 0.0
1	correlation	0.0	0.06 ± 0.02	0.01 ± 0.01	0.08 ± 0.04
2	mae	0.2	0.0 ± 0.0	0.1 ± 0.0	0.1 ± 0.1
3	correlation	0.2	0.02 ± 0.02	0.03 ± 0.02	0.07 ± 0.04
4	mae	0.4	0.0 ± 0.0	0.0 ± 0.0	0.1 ± 0.1
5	correlation	0.4	0.05 ± 0.02	0.03 ± 0.02	0.05 ± 0.03
6	mae	0.6	0.0 ± 0.0	0.0 ± 0.0	0.1 ± 0.0
7	correlation	0.6	0.06 ± 0.01	0.02 ± 0.01	0.09 ± 0.05

.

Table S13: Difference between the metrics for OphPred with XGBoost head calculated using the sequences from one super kingdom and the metrics calculated using all the sequences from the test set for the random($\varepsilon = 0.0$) and homology splits.

	metric	homology threshold(ε)	Bacteria (superkingdom)	Eukaryota (superkingdom)	Archaea (superkingdom)
0	mae	0.0	0.0 ± 0.0	0.1 ± 0.0	0.1 ± 0.1
1	correlation	0.0	0.04 ± 0.02	0.02 ± 0.01	0.1 ± 0.06
2	mae	0.2	0.0 ± 0.0	0.0 ± 0.0	0.1 ± 0.1
3	correlation	0.2	0.05 ± 0.02	0.04 ± 0.01	0.08 ± 0.04
4	mae	0.4	0.0 ± 0.0	0.1 ± 0.0	0.1 ± 0.1
5	correlation	0.4	0.06 ± 0.03	0.03 ± 0.02	0.12 ± 0.06
6	mae	0.6	0.0 ± 0.0	0.0 ± 0.0	0.1 ± 0.0
7	correlation	0.6	0.06 ± 0.02	0.02 ± 0.01	0.09 ± 0.04

.

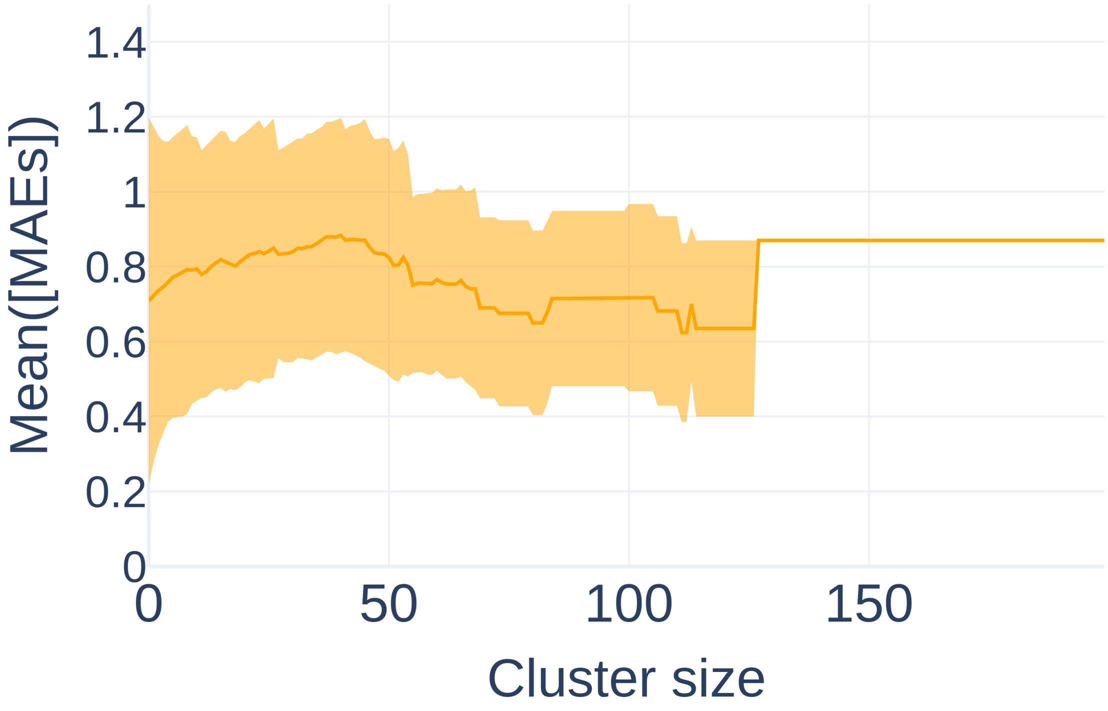

Figure S1: Mean of mean absolute errors on PFAM hold-outed clusters w.r.t to cluster size for the XGBoost model.

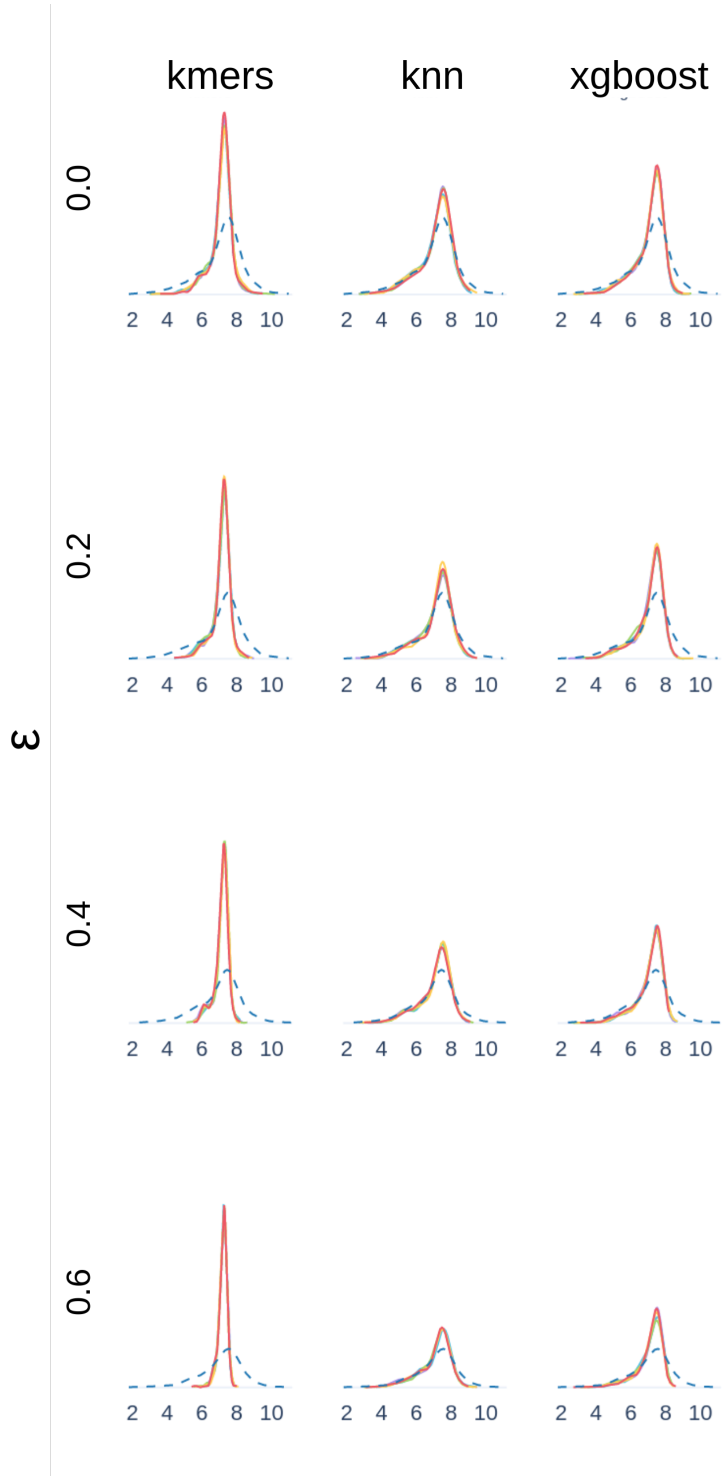

Figure S2: Histograms of predicted and true pH value for validation subset. Different colors stand for different random seeds. Solid and dashed lines indicate predicted and true pH values, respectively.

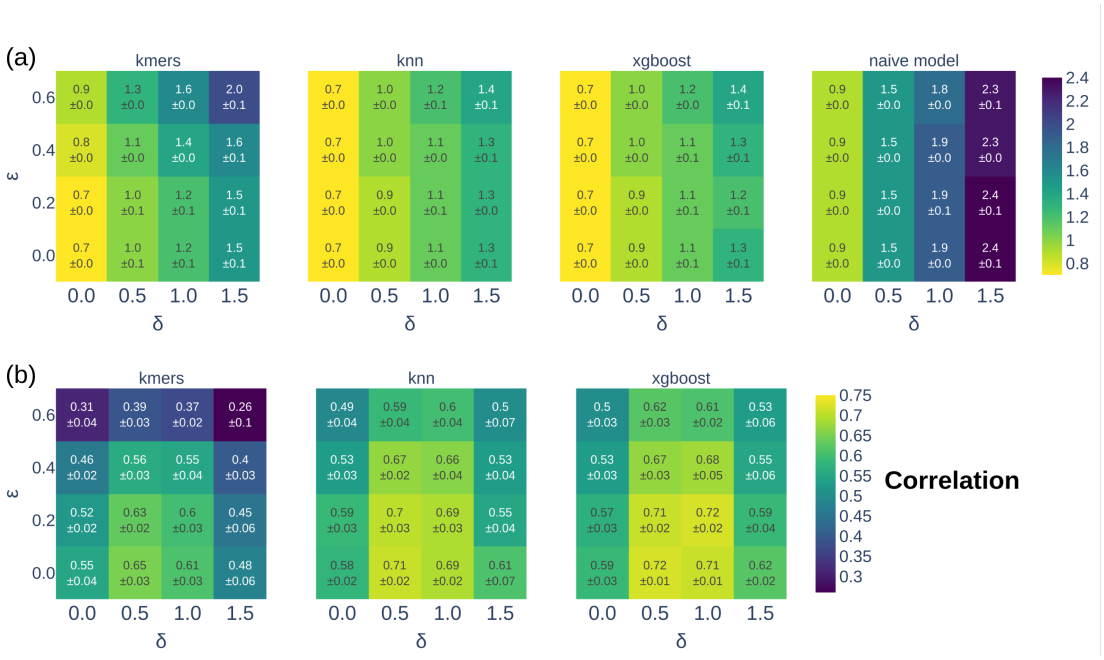

Figure S3: Heatmaps indicating mean absolute error(panel (a)) and correlation(panel (b)) for different models on different splits. The naive model always predicts the median value learned from a training set.

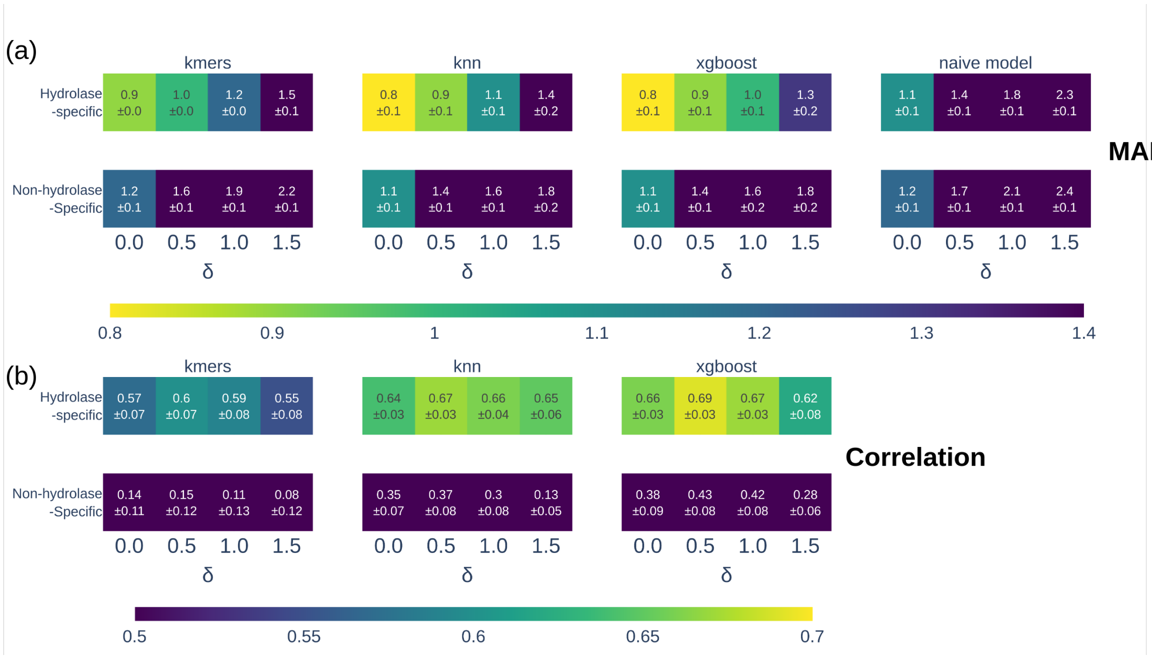

Figure S4: Heatmaps indicating mean absolute error(panel (a)) and correlation(panel (b)) for and hydrolase- and non-hydrolase-specific models. The naive model always predicts the median value learned from a training set.

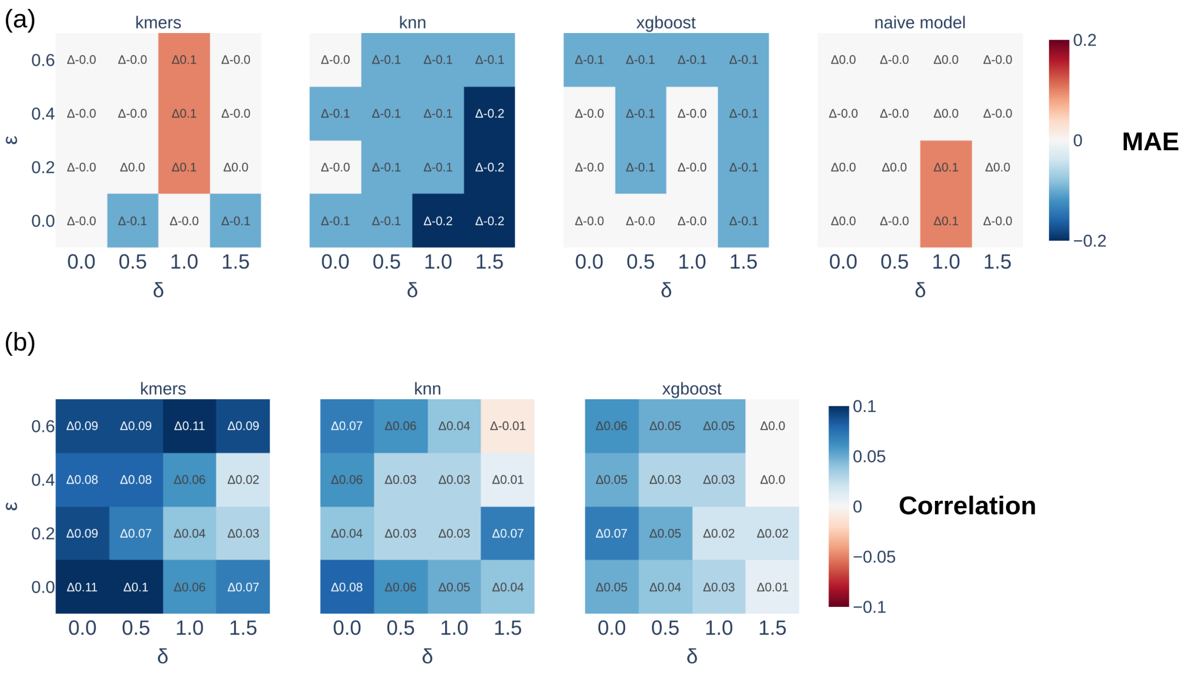

Figure S5: Heatmaps indicating difference of mean absolute errors(panel (a)) and correlations(panel (b)) for different models on homology splits after and before enrichment. The naive model always predicts the median value learned from a training set.

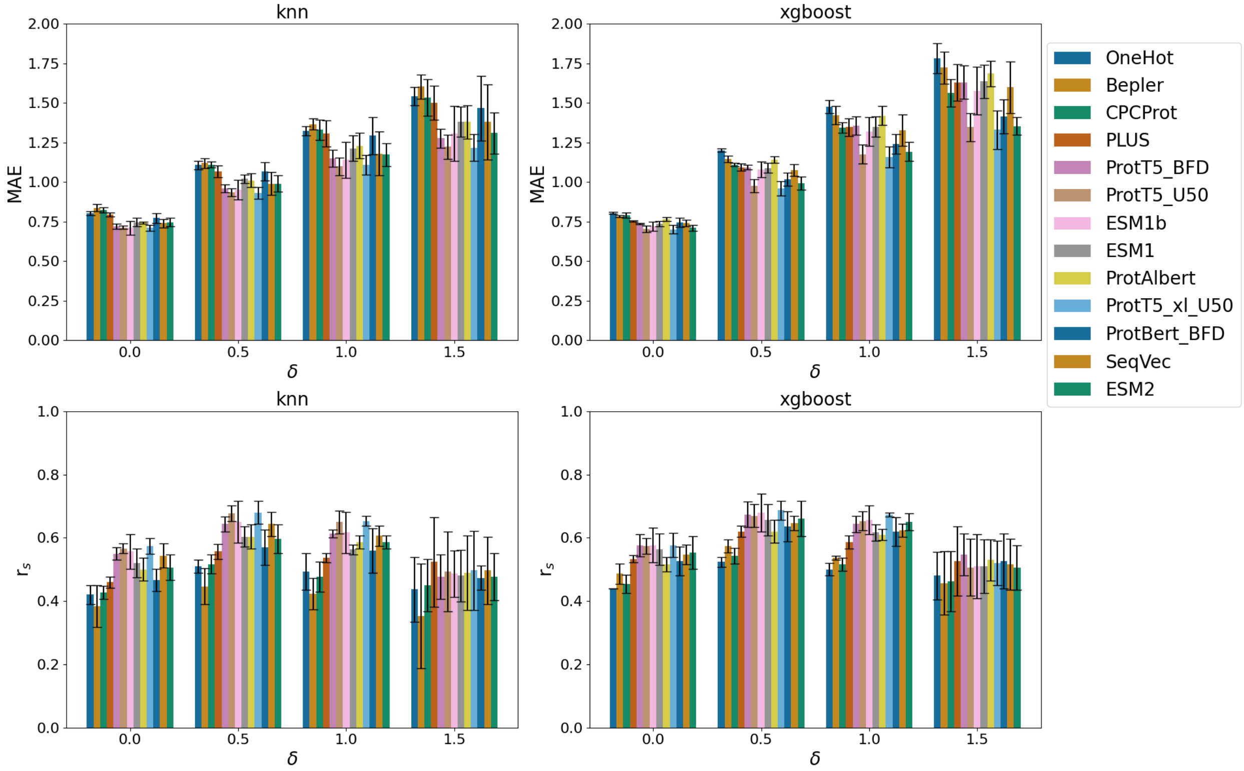

Figure S6: The performance metrics (top row - MAE, bottom row - correlation coefficient) calculated for models based on different embeddings on homology split($\varepsilon = 0.6$).

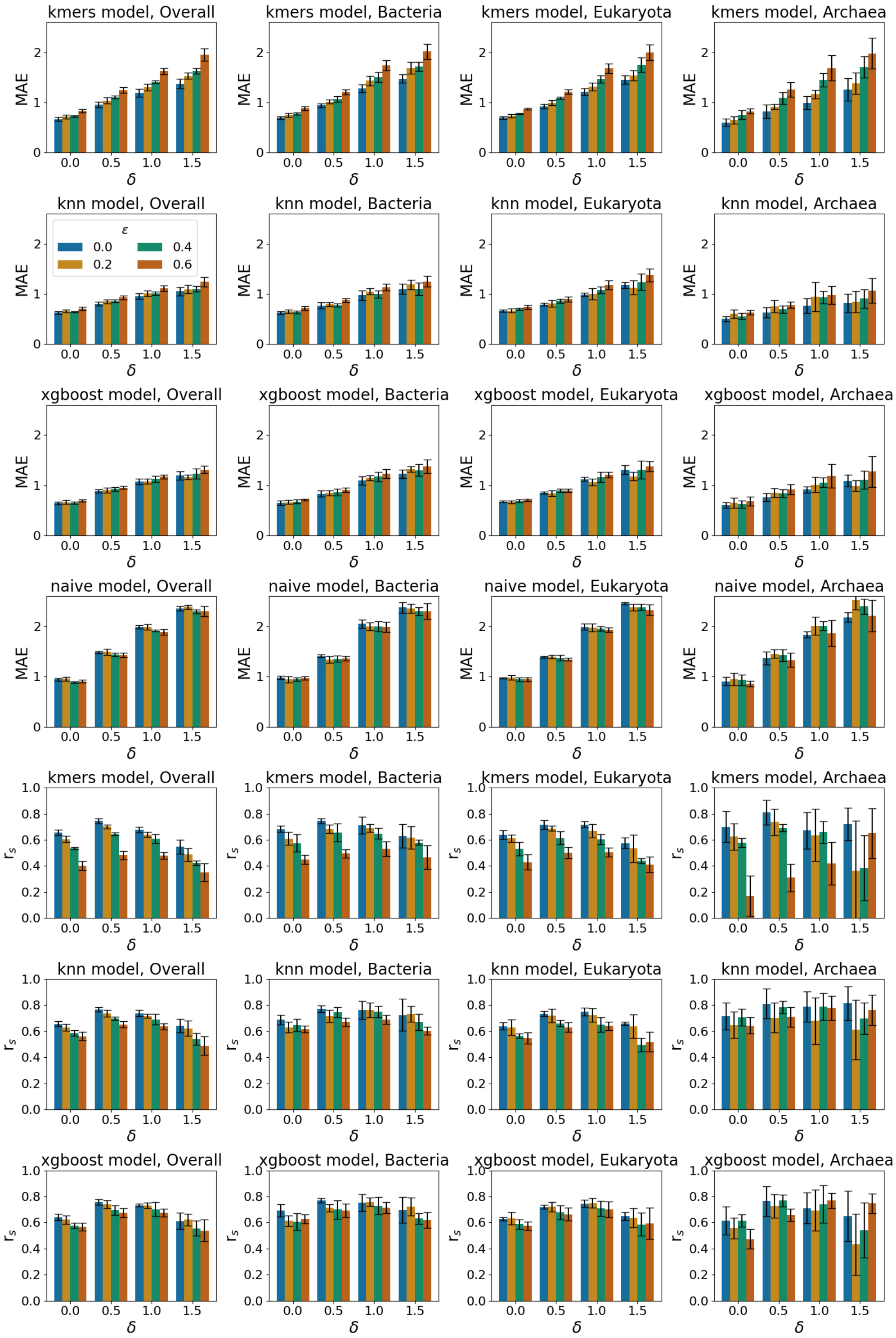

Figure S7: The performance metrics (top three rows - MAE, bottom three rows - correlation coefficient) for different models trained with the homology split ($\varepsilon = 0.6$). Columns correspond to the metric values calculated using all, bacterial, eukaryotic, and archaeal sequences from the test set, respectively.
